# Novel organisation and regulation of the *pic* promoter from enteroaggregative and uropathogenic *Escherichia coli*

**DOI:** 10.1080/21505594.2022.2111754

**Published:** 2022-08-16

**Authors:** Munirah M. Alhammadi, Rita E. Godfrey, Joseph O. Ingram, Gurdamanjit Singh, Camilla L. Bathurst, Stephen J.W. Busby, Douglas F. Browning

**Affiliations:** a Institute of Microbiology and Infection, School of Biosciences, University of Birmingham, Birmingham, UK; b Biology Department, Princess Nourah bint Abdulrahman University, Riyadh, Saudi Arabia; c College of Health & Life Sciences, Aston University, Birmingham, UK

**Keywords:** Bacterial gene regulation, CRP, enteroaggregative *Escherichia Coli* (EAEC), Fis, mucin, Pic, uropathogenic E. *coli* (UPEC), virulence

## Abstract

The serine protease autotransporters of the *Enterobacteriaceae* (SPATEs) are a large family of virulence factors commonly found in enteric bacteria. These secreted virulence factors have diverse functions during bacterial infection, including adhesion, aggregation and cell toxicity. One such SPATE, the Pic mucinase (protein involved in colonisation) cleaves mucin, allowing enteric bacterial cells to utilise mucin as a carbon source and to penetrate the gut mucus lining, thereby increasing mucosal colonisation. The *pic* gene is widely distributed within the *Enterobacteriaceae*, being found in human pathogens, such as enteroaggregative *Escherichia coli* (EAEC), uropathogenic *E. coli* (UPEC) and *Shigella flexneri* 2a. As the *pic* promoter regions from EAEC strain 042 and UPEC strain CFT073 differ, we have investigated the regulation of each promoter. Here, using *in vivo* and *in vitro* techniques, we show that both promoters are activated by the global transcription factor, CRP (cyclic AMP receptor protein), but the architectures of the EAEC and the UPEC *pic* promoter differ. Expression from both *pic* promoters is repressed by the nucleoid-associated factor, Fis, and maximal promoter activity occurs when cells are grown in minimal medium. As CRP activates transcription in conditions of nutrient depletion, whilst Fis levels are maximal in nutrient-rich environments, the regulation of the EAEC and UPEC *pic* promoters is consistent with Pic’s nutritional role in scavenging mucin as a suitable carbon source during colonisation and infection.

## Introduction

Many bacteria use the regulation of transcript initiation as a key strategy to control the expression of their genes, and it is generally accepted that the evolution of this regulation is an essential contributor to bacterial fitness, as they acquire the ability to colonise different habitats. Genomic studies have shown that bacterial pathogens have acquired many genes that encode specific virulence determinants, which facilitate colonisation of their hosts [1,2]. The expression of these genes is tightly regulated at the level of transcription, involving the interplay of transcription factors. Many common pathogens possess a virulence-associated regulon whose transcription is controlled by a “dedicated” transcription factor [1,2]. Such factors appear to have been recruited and adapted specifically to control the expression of virulence genes. This appears to be the case in strains of enteroaggregative *Escherichia coli* (EAEC), an important human pathogen that causes diarrhoea in adults and children and is endemic both in industrialized and developing countries [3–6]. Here, the AggR transcription factor activates the expression of dozens of virulence determinants, *e.g*. the attachment adherence fimbriae (AAF) required for colonization, the anti-aggregative protein dispersin (Aap) and its dedicated secretion system [7–11], and, thus, AggR is the master controller of virulence in these strains [10–13]. However, expression of some EAEC toxins and secreted proteins, such as the plasmid-encoded toxin (Pet), enteroaggregative heat-stable toxin (EAST-1), *Shigella* enterotoxin 1 (ShET1) and the Pic mucinase (protein involved in colonisation), are not controlled by AggR [10,13,14].

The Pet toxin, a cytotoxic autotransporter protein, which is secreted during infection by EAEC strain 042 [15,16], belongs to a large family of secreted virulence factors (the serine protease autotransporters of the *Enterobacteriaceae* (SPATEs)), commonly found in enteric bacteria [17]. Expression of Pet is positively controlled by the cyclic AMP receptor protein (CRP) and the nucleoid-associated protein Fis (factor for inversion stimulation) [18,19]. CRP is a global transcription factor whose activity is triggered by 5`-3` cyclic AMP (cAMP), an effector that signals depletion of certain nutrients and stress [20,21]. CRP functions as a dimer, with a helix-turn-helix motif in each subunit binding to adjacent major grooves at its DNA target [22,23]. The consensus base sequence for binding is 5`-TGTGANNNNNNTCACA-3` and, upon binding, at many targets, CRP interacts directly with RNA polymerase holoenzyme (RNAP), resulting in CRP-dependent activation of transcription by RNAP recruitment. CRP can make two distinct types of activatory interaction with RNAP [22,23]. In Class I activation, a surfaceexposed determinant on one subunit of the CRP dimer, Activating Region 1 (AR1), contacts a complementary determinant in the C-terminal domain of the RNAP alpha (α) subunit. In this instance, the DNA site for CRP is often located 49 base pairs upstream from the promoter −10 element (5`TATAAT-3`) [22–24]. In Class II activation, CRP usually binds 29 base pairs upstream of the −10 element and, additionally, contacts the *N*-terminal domain of the RNAP α subunit, with surfaceexposed determinant Activating Region 2 (AR2) [22–24]. In all *E. coli* strains studied so far, transcript initiation at hundreds of promoters is activated by CRP, functioning either by the Class I or Class II mechanism.

Like Pet, the Pic mucinase is a SPATE, and was originally identified in EAEC strain 042 and *Shigella flexneri* 2a, but, later, discovered in Uropathogenic *E. coli* (UPEC), and found to be distributed widely within the *Enterobacteriaceae* [17,25,26]. The Pic mucinase cleaves mucin, allowing EAEC both to use this substrate as a carbon source, and, to penetrate the mucus lining in the gut, increasing colonisation, and stimulating mucin secretion [25–30]. In addition, this important SPATE cleaves a wide range of substrates, including complement proteins, human leukocyte surface glycoproteins, and coagulation factor V, implicating Pic in bacterial serum resistance, haemagglutination and immunomodulation [25,26,31–33]. Here, we report the cloning and characterisation of the *pic* gene regulatory region from EAEC strain 042 and report that *pic* transcription is activated by CRP, using a Class I mechanism. We also investigated the *pic* promoter from UPEC strain CFT073, showing that it is also activated by CRP, but despite striking base sequence similarities with the EAEC *pic* promoter, activation is predominantly via a Class II mechanism. We show that both promoters have unusual features, and that CRP-dependent activation is counteracted by the global transcription factor Fis. We argue that what we observe is the result of evolutionary processes whereby transcription units are recruited into the regulons of different transcription factors.

## Materials and methods

### Bacterial strains, plasmids and DNA fragments

Table S1 describes the different *E. coli* strains that we employed in this study, together with details of plasmids and DNA fragments carrying promoters. Table S2 gives details of synthetic oligodeoxynucleotide primers that we used to amplify or to alter promoter-carrying DNA fragments Throughout, standard molecular biology procedures were used [34]. Each cloned promoter DNA fragment carries flanking EcoRI and HindIII sites to facilitate cloning and DNA sequences are numbered from the transcript start (designated as +1) as defined by the 5` RACE and potassium permanganate footprinting experiments. Upstream and downstream promoter locations are designated ‘-‘ and “+,” respectively. Base pair substitutions in both the UPEC and EAEC *pic* promoters are designated pNX, where N is the substitution’s position relative to the transcript start and X is the substitution in the non-template strand. As a source of DNA for EMSA (electromobility shift assays) and footprinting procedures, promoter fragments were cloned into pSR [35]. To determine promoter activity, each promoter fragment was cloned into the *lac* expression vectors pRW224 and pRW225 to generate either a *lacZ* transcriptional or translational fusion, respectively, [36]. Derivatives of pSR were selected for using media supplemented with 100 μg ml^−1^ ampicillin, whilst for pRW224 and pRW225 constructs, 15 μg ml^−1^ tetracycline was used.

### DNA construction

The EAEC *pic*p042 and UPEC *pic*p073 promoter fragments were amplified by PCR using primers in Table S2, with EAEC 042 and UPEC CFT073 genomic DNA, respectively, as template. Flanking EcoRI and HindIII sites facilitated cloning into different plasmids. To introduce the p37A and p34T mutations in the UPEC *pic* promoter, the *pic*p073 fragment was amplified by PCR, using either primer picCTFp37A or picCTFp34T and primer lacZRev, with pRW224/*pic*p073 as template. Products were cloned into pRW224 and verified by DNA sequencing.

Point mutations were introduced into the putative −10 elements of *pic*p042 and *pic*p073 using megaprimer PCR [37]. During the first round of PCR, megaprimers were generated using primers *pic*p042-UKO, *pic*p073-UKO, *pic*p042-DKO or *pic*p073-DKO with primer lacZRev and plasmids pRW224/*pic*p042 or pRW224/*pic*p073 as template. In the second round of PCR, purified megaprimer was used with primer D10520 and various pRW224/*pic*p042 or pRW224/*pic*p073 constructs as template. All DNA fragments were restricted with EcoRI and HindIII and cloned into pRW224.

The *pic*p042 and *pic*p073 promoter fragments were cloned into pRW225 to generate *lacZ* translational fusions. Fragment DNA was PCR amplified using either primers pic042–225 or pic073–225 with D10520 and pRW224/*pic*p042 or pRW224/*pic*p073 as template. PCR products were cut with EcoRI and HindIII and cloned into pRW225. All constructs were confirmed using Sanger DNA sequencing.

### Error-Prone PCR of the EAEC *pic*p042 promoter fragment

To introduce random substitutions throughout the EAEC *pic*p042 promoter fragment, error-prone PCR was used. Primers D10520 and lacZRev were used with pRW224/*pic*p042 as template with standard *Taq* DNA polymerase. PCR products were restricted with *Eco*RI and *Hin*dIII and cloned into pRW224. *E. coli* K-12 BW25113 cells were transformed with the resultant plasmid DNA and plated onto MacConkey agar plates, containing tetracycline. Colonies, in which the intensity of the Lac+ phenotype had decreased, were selected. Plasmid DNA was purified, and the sequence of *pic* promoter fragments obtained using Sanger Sequencing.

### 5` RACE (rapid amplification of cDNA ends)

Overnight cultures of BW25113 cells, containing either pRW224/*pic*p042 or pRW224/*pic*p073 were grown in LB medium at 37 °C with shaking until an OD_600_ = 1. Total RNA was isolated using an Isolate II RNA Mini Kit (Meridian Bioscience), as specified by the manufacturers. Specific mRNA was then converted to cDNA using primer SP1 and a 5`Race kit 2^nd^ Generation (Roche), according to the manufacturer’s instructions. Strand specific cDNA was A-tailed on the 3` end and then amplified by PCR using primers dT-anchor and lacZRev. PCR products were then purified and cloned into pJET1.2, using the Clone JET PCR Cloning Kit (Thermo Scientific), as specified by the manufacturers. Constructs were sequenced using Sanger Sequencing.

### Proteins

Purified RNAP was obtained from New England Biolabs (Ipswich, MA, USA). Fis protein was purified as in Osuna *et al*. [38]. CRP over-expression and purification was carried out as described in Ghosaini *et al* [39].

### Electromobility shift assays (EMSA)

EMSA assays using purified Fis were carried out as detailed in Godfrey *et al*. [40]. Purified *pic* promoter fragments were prepared from pSR based vectors and end labelled with [γ-^32^P]-ATP. In each reaction, 0.5 ng of each ^32^P labelled fragment was incubated with different amounts of Fis protein. The composition of the EMSA reaction buffer was 20 mM HEPES (pH 8.0), 5 mM MgCl_2_, 50 mM potassium glutamate, 1 mM DTT, 25 μg ml^−1^ herring-sperm DNA, 0.5 mg ml^−1^ BSA and 5% (v/v) glycerol (10 μl final volume). Samples were incubated at 37°C for 20 min, and then immediately separated on a 6% polyacrylamide gel (12 V cm^−1^), containing 0.25 x TBE and 2% glycerol. Dried gels were analysed with a Bio-Rad Molecular Imager FX and Quantity One software (Bio-Rad).

## Potassium permanganate footprinting experiments

Potassium permanganate footprinting experiments were carried out on ^32^P-end-labelled *pic* promoter fragments, as in Browning *et al*. [41]. Reactions contained 1.35 nM template DNA in 20 mM HEPES (pH 8.0), 50 mM potassium glutamate, 5 mM MgCl_2_, 1 mM DTT, 0.5 mg ml^−1^ BSA and 200 µM cAMP (final volume 20 µl). 50 nM *E. coli* RNA polymerase (NEB) was added to reactions, where indicated. Samples were analysed using denaturing gel electrophoresis and calibrated with Maxam-Gilbert “G+A” sequencing reactions. Gels were visualised using Quantity One software (Bio-Rad) and a Bio-Rad Molecular Imager FX.

### Assays of *pic* promoter activity

To analyse expression from the *pic* promoter, DNA fragments were cloned into the pRW224 and pRW225 *lac* expression vectors, and each construct was transferred into various *E. coli* K-12 Δ*lac* cells (Table S1). β-galactosidase activity was determined using a Miller [42] protocol as in [43]. Cells were cultured in LB medium with shaking at 37°C until they reached mid-logarithmic phase (OD_650_ = 0.40.6). In all cases, βgalactosidase activities are stated as nmol of ONPG hydrolysed min^−1^ mg^−1^ dry cell mass and activities are the average of at least three independent biological replicates and average values are reported.

### Isolation of the Pic protein from the extracellular medium

To examine the secretion of the Pic mucinase into the extracellular medium, *E. coli* K12 BW25113 cells and its isogenic Δ*crp* and Δ*fis* derivatives were transformed with plasmid pPic, which carries the EAEC *pic* gene and promoter region [27]. Cultures were grown overnight with shaking at 37°C in 50 ml of LB medium, supplemented with tetracycline. The OD_600_ of each culture was normalized to allow secreted protein levels to be compared, and cells were pelleted by centrifugation at 10,000 x g for 10 minutes at 4°C. Supernatants, which contain the secreted Pic protein, were filtered through 0.22 µm filters (Millipore) and proteins were precipitated by adding 10% (w/v) trichloroacetic acid (Fisher Scientific), as in our previous work [44]. Secreted Pic protein were analysed using SDS-PAGE and stained with Coomassie blue.

### Modelling of mRNA secondary structure

Secondary structure prediction of the EAEC and UPEC *pic* mRNA transcripts was carried out using Mfold [45] .

## Results and discussion

### The Pic promoter from EAEC strain 042 is activated by CRP

**Figure 1.**
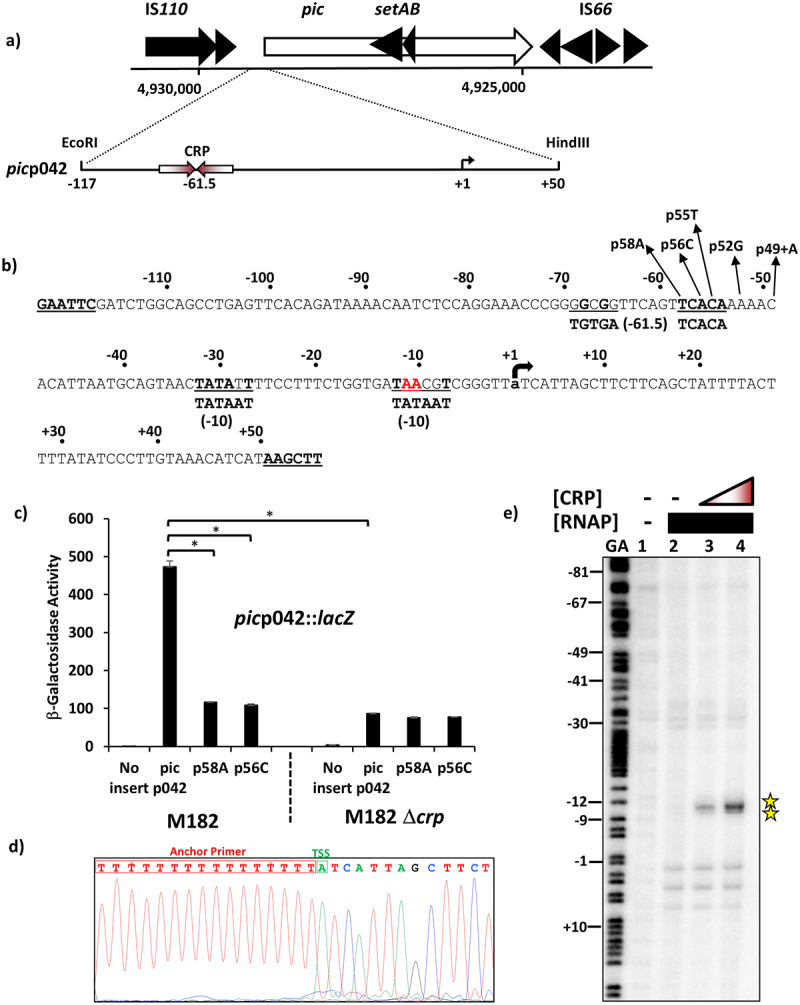
CRP-Dependent activation of the EAEC 042 *pic* promoter. (a) the panel shows the organisation of the *pic* locus on the EAEC 042 chromosome, detailing the flanking IS*110* and IS*66* family elements. The exploded view shows a schematic representation of the *pic*p042 promoter fragment. The CRP binding site is shown as inverted arrows and the start site of transcription (+1) is indicated by a bent arrow. The flanking EcoRI and HindIII sites used to clone the fragment are indicated. (b) the base sequence of the *pic*p042 promoter fragment from EAEC 042. The CRP-binding site and two proposed −10 promoter elements are underlined and matches to their respective consensus sequences are in bold [23,24]. The transcription start site (+1) is lower case and indicated by a bent arrow and substitutions, which change the CRP-binding site and adjacent sequences, are shown. The bases that were identified by potassium permanganate footprinting as being single stranded in the open complex are highlighted in red. Terminal EcoRI and HindIII sites are bold and underlined. (c) the panel details β-galactosidase activities determined in the Δ*lac E. coli* K-12 strain M182 and its *δcrp* derivative. Cells carried the *lac* expression vector pRW224 into which EAEC 042 *pic*p042 promoter fragments were cloned. The p58A and p56C substitutions disrupt the CRP binding site at the *pic* promoter, see (b). Cells were cultured in LB medium and βgalactosidase activities are stated as nmol of ONPG hydrolysed min^−1^ mg^−1^ dry cell mass, activities are the average of at least three independent biological replicates. Standard deviations are shown and * indicates *P* < 0.01 using a Student’s *t*-test. (d) the panel shows the sequence trace from a 5` RACE experiment, which determined the EAEC 042 *pic* promoter transcription start site (TSS: green box). (e) End-labelled *pic*p042 AatII-HindIII fragment was incubated with RNA polymerase and CRP and subjected to potassium permanganate footprinting. The concentration of CRP was as follows: lanes 1 and 2, no protein; lane 3, 400 nM; lane 4, 800 nM. Each reaction contained 50 nM RNA polymerase and 200 µm cAMP. Maxam-Gilbert ‘G+A’ sequencing reactions have been run to calibrate the gel. The location of cleavage sites within the EAEC 042 *pic* promoter are highlighted by stars (and in red in panel (b)).

To investigate the regulation of Pic mucinase expression, we inspected the DNA base sequence immediately upstream of the translation start of the annotated *pic* gene on the EAEC strain 042 chromosome. Using PCR with synthetic oligodeoxynucleotide primers, we amplified the *pic*p042 fragment, which carries the 167 base pair sequence, immediately upstream from the Shine-Dalgarno sequence of the *pic* gene, flanked by an upstream EcoRI and a downstream HindIII site (Figure 1(a,b)). This was then cloned into pRW224, a well-characterised low copy number *lac* expression vector such that any promoter in the fragment will drive *lac* gene expression [36] . Colonies of *Δlac E. coli* K-12 strain M182 carrying pRW224 with the *pic*p042 fragment scored as red Lac+ on MacConkey lactose indicator plates, whereas *Δlac* strain M182 carrying pRW224 with the starting short linker fragment scored as white Lac-.

In a preliminary experiment, we checked that longer fragments gave similar expression, and searched for base changes in the *pic*p042 fragment that reduced expression. To do this, we amplified the *pic*p042 fragment using error-prone PCR, cloned the resulting product into pRW224 and transformed plasmids into M182 *Δlac* cells, plating transformants on MacConkey lactose plates. The base sequence of the *pic*p042 fragment was then determined from colonies that appeared pale pink rather than red Lac+. The first mutations that we found by this method were located 50 bp from the EcoRI end of the fragment (Figure 1b), and we noted that this region carries the sequence element 5`-GGCGGTTCAGTTCACA-3`, which is a 7/10 match to the well-established consensus DNA target for the cyclic AMP receptor protein (CRP) [22,23]. Hence, we concluded that the *pic*p042 fragment carries the EAEC 042 *pic* promoter, and used the pRW224 *lac* gene expression vector to measure its activity and investigate its dependence on CRP. Figure 1(c) illustrates levels of βgalactosidase activity, measured in the M182 *Δlac* host, and its *Δcrp* derivative, carrying pRW224 with the starting *pic*p042 fragment, as well as derivatives carrying the p58A or p56C mutations that alter key bases in the suggested DNA site for CRP (Figure 1b). The data show that the *pic*p042 fragment carries one or more CRP-dependent promoters.

Previous research has shown that, for most CRP-activated promoters in *E. coli*, the promoter 10 hexamer element is located either 29 or 49 base pairs downstream from the centre of the DNA site for CRP [23]. Inspection of the *pic*p042 sequence shows good matches to the consensus 10 hexamer sequence (5`-TATAAT-3`) at both locations: 5`-TATATT-3` and 5`-TAACGT-3`, located 29 and 49 base pairs downstream, respectively (Figure 1b). Hence, we experimentally determined the transcript start point by sequencing the 5` end of RNA extracted from growing cells (Figure 1d) and used an *in vitro* potassium permanganate unwinding assay to measure the location of CRP-induced unwinding in the *pic*p042 fragment (Figure 1e). Figure 1d illustrates the result of primer extension analysis of the 5` end of the RNA transcript that initiates in the *pic*p042 fragment, after amplification using the 5`RACE method (rapid amplification of cDNA ends). The experiment shows that transcription in the *pic*p042 fragment starts 61 base pairs downstream from the centre of the DNA site for CRP and suggests that the functional 10 element is 5`-TAACGT-3`. This was confirmed by the *in vitro* experiment illustrated in Figure 1e, where potassium permanganate was used to detect local CRP-dependent unwinding. In brief, the purified fragment was 5`-end labelled with ^32^P at the HindIII end and incubated with purified *E. coli* RNA polymerase holoenzyme, either with or without cAMP and CRP. Permanganate modifies single-stranded thymine residues and results in strand cleavage that is detected by polyacrylamide gel analysis [46]. The data in Figure 1e show that CRP drives unwinding of bases in the 5`-TAACGT-3` hexamer, located 49 base pairs downstream of the centre of the DNA site for CRP. These results argue that transcription of the EAEC strain 042 *pic* gene is primarily driven by a Class I CRP-dependent promoter.

### The UPEC strain CFT073 *picU* promoter is also activated by CRP

**Figure 2.**
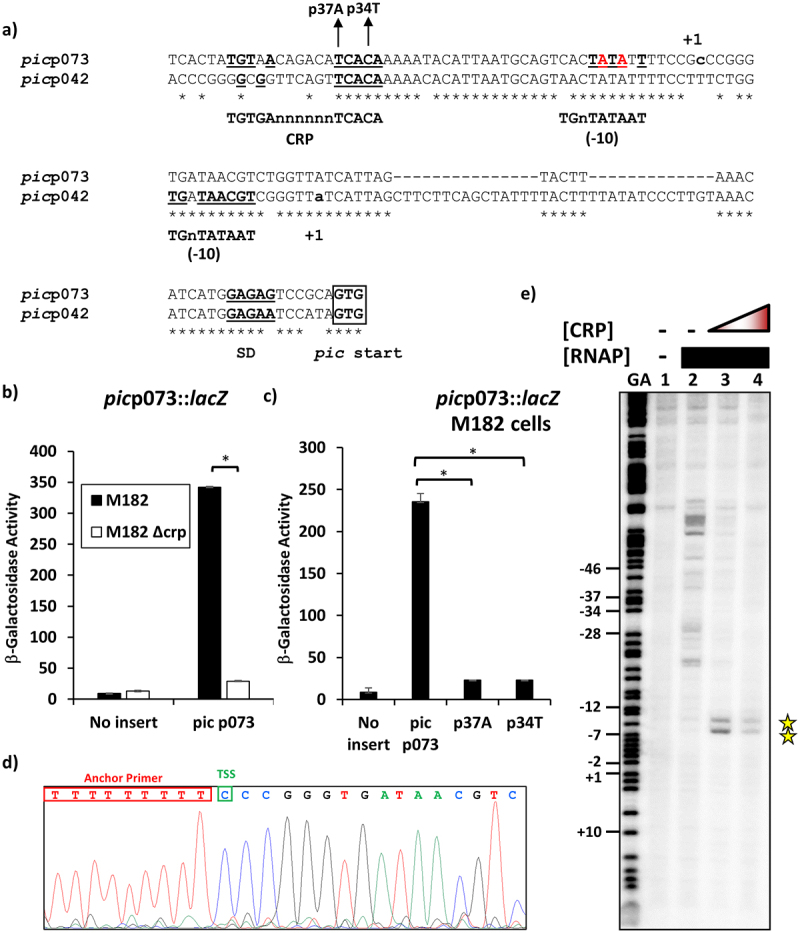
CRP-Dependent activation of the UPEC CFT073 *picU* promoter. (a) the panel shows an alignment of the UPEC *pic*p073 promoter sequence with the sequence from EAEC *pic*p042. The CRP-binding site and −10 promoter elements are underlined, with matches to their respective consensus sequences in bold [23,24]. For both fragments, transcription start sites (+1) are lower case bold, translation initiation codons (GTG) are boxed and the Shine–Dalgarno sequences (SD) are bold and underlined. The position of the p37A and p34T substitutions, which disrupt the CRP-binding site in the UPEC *pic*p073 promoter fragment, are shown and the bases, identified by potassium permanganate footprinting as being single stranded in the open complex, are in red. (b) the panel details β-galactosidase activities determined in the Δ*lac E. coli* K-12 strain M182 and its *δcrp* derivative. Cells carried the *lac* expression vector pRW224 into which the UPEC *pic*p073 promoter fragment was cloned. (c) the panel desplays β-galactosidase activities determined in the strain M182, with cells carrying various UPEC *pic*p073 promoter fragments, cloned into pRW224. The p37A and p34T substitutions disrupt the CRP binding site within the *pic*073 promoter fragment, see (a). In both (b) and (c), cells were cultured in LB medium and β-galactosidase activities are the average of at least three independent biological replicates. Standard deviations are shown and * indicates *P* < 0.01 using a Student’s *t*-test. (d) the panel shows the sequence trace from a 5` RACE experiment, which determined the UPEC *picU* promoter transcription start site (TSS: green box). (e) End-labelled *pic*p073 AatII-HindIII fragment was incubated with RNA polymerase and CRP and subjected to potassium permanganate footprinting. The concentration of CRP was as follows: lanes 1 and 2, no protein; lane 3, 400 nM; lane 4, 800 nM. Reactions contained 50 nM RNA polymerase and 200 µm cAMP and Maxam-Gilbert ‘G+A’ sequencing reactions have been included. The location of potassium permanganate cleavage sites are shown starred.

The paradigm UPEC strain CFT073 also secretes the Pic mucinase (PicU), which is expressed during colonisation of the bladder and plays a role during systemic infection [26,47,48]. Inspection of the base sequence upstream of the UPEC *picU* translation start revealed many similarities to the corresponding *pic* gene of EAEC 042. Strikingly, this region carries the sequence element 5`-TGTAACAGACATCACA-3`, which is a 9/10 match to the consensus DNA target for CRP (Figure 2a) [23]. Thus, again using PCR and synthetic oligodeoxynucleotide primers, we amplified the EcoRI-HindIII *pic*p073 fragment, carrying UPEC CFT073 sequence immediately upstream of the *picU* Shine-Dalgarno sequence, and cloned it into pRW224. As expected, colonies of M182 carrying pRW224 containing the *pic*p073 fragment scored as red Lac+ on MacConkey lactose plates. Figure 2(b) illustrates levels of βgalactosidase activity, measured in the M182 *Δlac* host, and its *Δcrp* derivative, carrying pRW224 with the starting *pic*p073 fragment, whilst Figure 2(c) shows the effect of derivatives carrying either the p34T or the p37A mutations that alter key bases in the suggested DNA site for CRP. The data show that the *pic*p073 fragment carries one or more CRP-dependent promoters. Remarkably, the *pic*p073 sequence, like *pic*p042, also contains hexamer elements with a good match to the consensus 10 hexamer (5`TATAAT3`) at both 29 base pairs and 49 base pairs downstream from the centre of the DNA site for CRP (respectively, 5`TATATT-3` and 5`-TAACGT-3`). Hence, again, we experimentally determined the transcript start point in growing cells (Figure 2d) and used *in vitro* potassium permanganate footprinting to measure the location of CRP-induced unwinding in the *pic*p073 fragment (Figure 2e). The data in Figure 2(d) show that transcription in the *pic*p073 fragment starts 40 base pairs downstream from the centre of the DNA site for CRP, suggesting that the functional 10 element must be 5`-TATATT-3`. This is confirmed by the *in vitro* potassium permanganate footprinting experiment illustrated in Figure 2(e), with CRP directing the unwinding of bases in the 5`-TATATT-3` hexamer, located 29 base pairs downstream of the centre of the DNA site for CRP. These results argue that transcription of *picU* in UPEC CFT073 is primarily driven by a Class II CRP-dependent promoter.

### Similarities and differences between the EAEC 042 and UPEC CFT073 *pic* promoters

**Figure 3.**
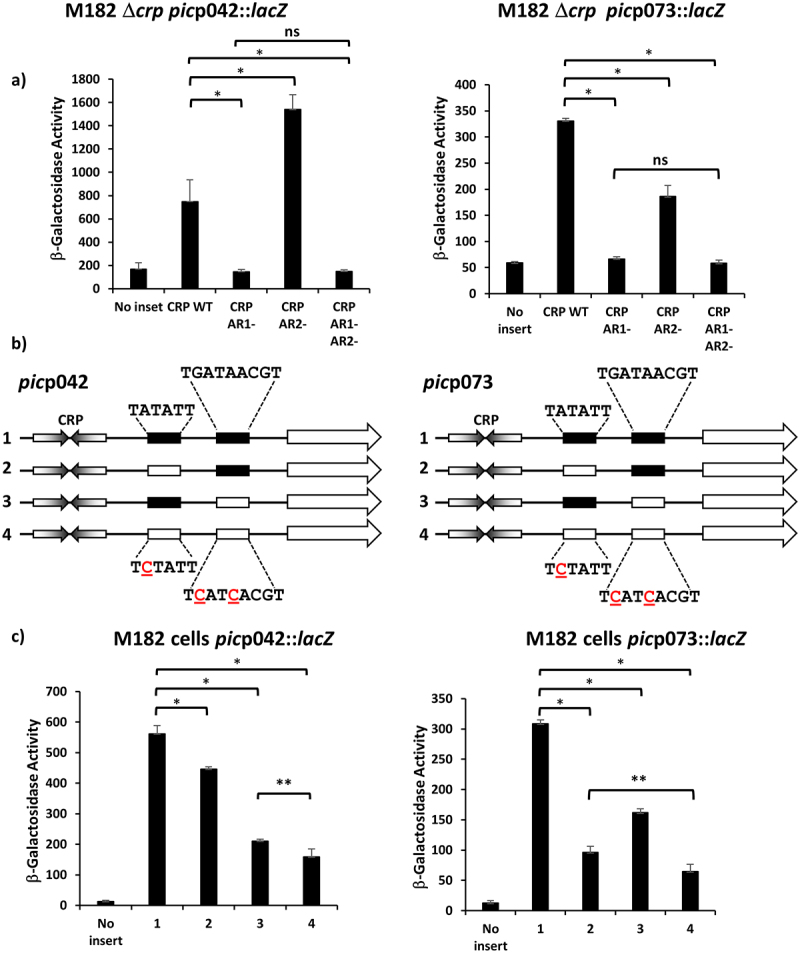
The EAEC and UPEC *pic* promoters use different −10 elements. (a) the panel details β-galactosidase activities from *E. coli* strain M182 *Δcrp*, carrying the *lac* expression vector pRW224 into which the EAEC *pic*p042 or UPEC *pic*p073 promoter fragments were cloned. Cells also carried either plasmid pDCRP or derivatives of pDCRP, encoding substitutions in CRP activating regions AR1 (HL159) and/or AR2 (KE101). (b) the panel shows schematic representations of the wild type EAEC *pic*p042 and UPEC *pic*p073 promoter fragments and fragments carrying substitutions in putative upstream and downstream −10 promoter elements. CRP binding sites are shown as inverted arrows and wild-type and mutant −10 elements are shown as filled or empty boxes, respectively. (c) the panel displays β-galactosidase activities in *E. coli* strain M182, carrying pRW224 into which the EAEC *pic*p042 or UPEC *pic*p073 promoter fragments depicted in (b) were cloned. Note that the numbering of promoter inserts is the same in both (b) and (c). For all experiments, cells were grown in LB, βgalactosidase activities are stated as nmol of ONPG hydrolysed min^−1^ mg^−1^ dry cell mass and are the average of at least three independent biological replicates. Standard deviations are shown and * indicates *P* < 0.01, ** indicates *P* < 0.05 and ns (not significant) P > 0.05, using a Student’s *t*-test.

The activation of transcript initiation by CRP at target promoters is dependent on specific CRP-RNAP interactions involving AR1 at Class I promoters, and both AR1 and AR2 at Class II promoters [22,23]. To measure the effects of disrupting the different activating regions on *pic* promoter activity, we transformed the *lac* expression vector, pRW224, carrying either the *pic*p042 or *pic*p073 fragment into M182 *Δlac Δcrp* cells and then introduced a second plasmid, pD, carrying wild type CRP, or CRP carrying defective AR1, defective AR2 or defective AR1 and AR2. The data, illustrated in Figure 3a, indicate that, with the EAEC *pic*p042 fragment, disruption of AR1 prevents CRP-dependent activation, whilst disruption of AR2 increases activation. In contrast, with the UPEC *pic*p073 fragment, alteration of either AR1 or AR2 leads to a reduction in CRP-dependent activation.

In a second set of comparison experiments, we exploited previous findings that the activity of bacterial promoter −10 elements is reduced to near zero by alteration of the highly conserved A at the second position of the hexamer. Hence starting with either the *pic*p042 or *pic*p073 fragments cloned in pRW224, we inactivated either the upstream -10 element, or the downstream −10 element, or both (Figure 3b). Data in Figure 3(c) show that, with the *pic*p042 fragment, a large reduction in promoter activity is caused by inactivation of the downstream -10 element. In contrast, with the *pic*p073 fragment, the biggest reduction is observed when the upstream -10 element is mutated, although changing the downstream element does cause a significant reduction.

**Figure 4.**
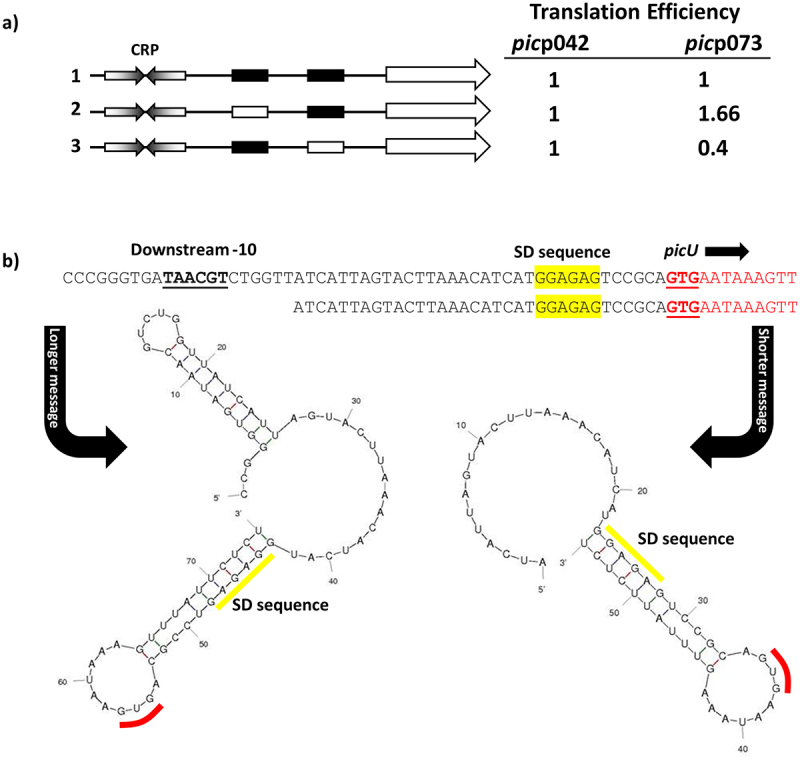
Translation efficiency differs for the EAEC *pic*p042 and UPEC *pic*p073 promoter fragments. (a) the panel shows schematic representations of the wild-type EAEC *pic*p042 and UPEC *pic*p073 promoter fragments and derivatives with substitutions in the upstream or downstream −10 promoter elements. CRP binding sites are shown as inverted arrows and wild-type and mutant −10 elements are shown as filled or empty white boxes, respectively. Fragments were cloned into pRW224 and pRW225 to generate lacZ transcription fusions and translation fusions, respectively. β-galactosidase activities were measured in *E. coli* M182 carrying either pRW224 or pRW225 into which the various EAEC *pic*p042 and UPEC *pic*p073 promoter fragments were cloned. Translation Efficiency is shown as the ratio of the values observed for pRW224 (transcription only) and pRW225 (transcription and translation) and is set as 1 for each starting wild type promoter. Cells were cultured in LB medium, βgalactosidase activities are the average of at least three independent values. (b) the panel shows the predicted secondary structure of the 5` ends of the mRNA when initiated from either the upstream or downstream -10 element in the UPEC *pic*p073 fragment. The location of the downstream −10 element, the Shine-Dalgarno sequence (SD) and translation initiation codon (GUG) are shown.

Taken together, our data suggest that CRP at the EAEC strain 042 *pic* promoter almost exclusively uses a Class I activation mechanism, but, at the UPEC CFT073 *picU* promoter, Class II activation predominates, though Class I activation is possible. Since, the consequence of initiation at one locus or another is to alter the 5`-end transcript sequence, we extended the HindIII end of each fragment to include the corresponding *pic* gene translation start, and cloned the resulting fragments into pRW225, a derivative of pRW224 that permits translation fusions, thereby fusing the *N*-terminal end of the *pic* open reading frame to the *lacZ* gene. Hence, for each fragment, we could measure activity due to transcription only (using the pRW224 derivatives), or due to transcription and translation (using the pRW225 derivatives), and differences in the ratio of the two values will reflect differences in translation, due to altered initiation or changes in transcript stability. With promoter fragments derived from *pic*p042, this ratio, within experimental error, remains constant, irrespective of the mutations in either −10 element (Figure 4a). In sharp contrast, with the fragments derived from *pic*p073, relative expression of the Pic-LacZ fusion protein is higher when Class II activation is prevented by mutation of the upstream -10 element, but is lower when Class II activation is optimised by mutation of the downstream −10 element, which results longer transcripts (Figure 4a). Inspection of secondary structures at the 5` end of transcripts that initiate in *pic*p073 (Figure 4b) suggests a simple reason for this, with the longer transcript, resulting from Class II activation, carrying an inhibitory 5` stem-loop structure that is absent from shorter transcripts. Note that, because promoter activity in the *pic*p042 fragment mostly uses the downstream -10 hexamer element, and because the EAEC strain 042 sequence contains two short insertions just upstream of the *pic* gene Shine-Dalgarno sequence (see Figure 2a), the lengths of the 5` untranslated sequences for the EAEC strain 042 and UPEC CFT073 genes are similar, but the EAEC *pic* transcript lacks the inhibitory structure.

## Repression of *pic* promoter activity by Fis

**Figure 5.**
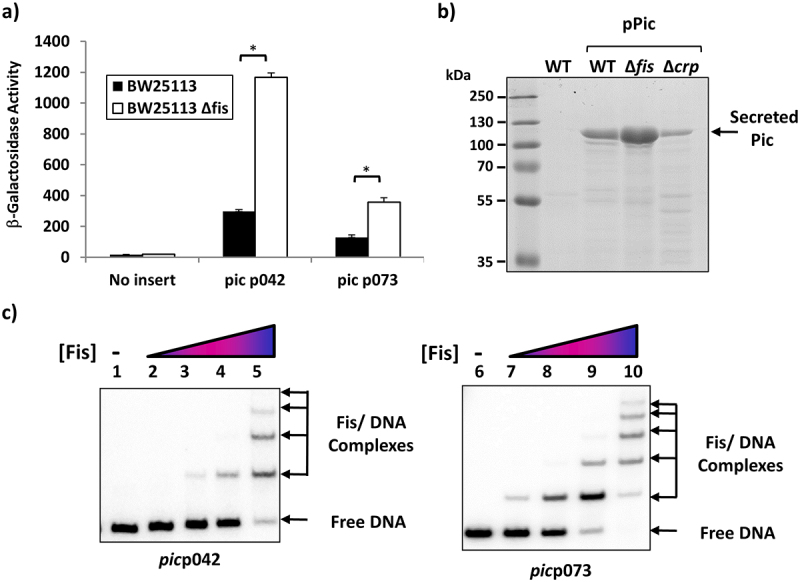
Fis represses the EAEC and UPEC *pic* promoters. (a) the panel illustrates β-galactosidase activities measured in the Δ*lac E. coli* K-12 strain BW25113 and its *δfis* derivative. Cells carried the *lac* expression vector pRW224 into which the EAEC 042 *pic*p042 or UPEC CFT073 *pic*p073 promoter fragments were cloned. Cells were grown in LB medium and βgalactosidase activities are the average of at least three independent biological replicates. Standard deviations are shown and * indicates *P* < 0.01 using a student’s *t*-test. (b) the panel shows an SDS-PAGE gel run to quantify the amount of Pic protein secreted into the external medium by wild-type strain BW25113 (WT) and its Δ*fis* and Δ*crp* derivatives. Where indicated, cells carried plasmid pPic, in which the EAEC 042 *pic* promoter and gene are cloned into plasmid pACYC184 [27]. The arrows indicate the location of secreted Pic protein (116 kDa). PageRuler prestained protein ladder (Fisher Scientific) were run to calibrated the gel. (c) Gel retardation assays of the EAEC *pic*042 and UPEC *pic*073 promoter fragments with purified Fis protein. End-labelled *pic* promoter fragments were incubated with purified Fis protein: lanes 1–5, *pic*p042 *Eco*RI-*Hin*dIII fragment; lanes, 6–10, *pic*p073 *Eco*RI-*Hin*dIII fragment. The amount of Fis protein in each reaction was: lanes 1 and 6, no protein; lanes 2 and 7, 200 nM; lanes 3 and 8, 400 nM; lanes 4 and 9, 800 nM; lanes 5 and 10, 1.35 µm. The location of Fis/DNA complexes and unbound DNA is indicated.

To our knowledge, the only other EAEC virulence factor regulated by CRP is the *pet* gene, which encodes an autotransporter-toxin. Expression of the EAEC strain 042 *pet* gene, and other related toxins in other strains, is co-activated by CRP and Fis, another global DNA-binding protein that functions to modulate transcript initiation at specific promoters [18,19]. Thus, *pet* gene expression is greatly reduced in *fis* mutant strains. To determine whether Fis plays any role at the *pic* gene promoter, we measured βgalactosidase activity in *fis*^*+*^ and *Δfis* cells containing pRW224, carrying either the *pic*p042 or *pic*p073 promoter fragment. Data illustrated in Figure 5(a) show that, with both fragments, promoter expression is higher in the *Δfis* background, suggesting that Fis functions as a repressor. To examine this further, *E. coli* K-12 BW25113 cells and *Δfis* and *Δcrp* isogenic derivatives were transformed with plasmid pPic, which carries the complete EAEC 042 *pic* transcription unit [27], and Pic protein secreted into the external medium was analysed by SDS PAGE (Figure 5b). The results confirm that CRP and Fis work positively and negatively, respectively, at the *pic* promoter. Furthermore, EMSAs, performed with the *pic*p042 fragment show that Fis binds to multiple targets, consistent with a role as a repressor, with similar results observed for the *pic*p073 fragment (Figure 5c).

## Conclusions

Bacterial transcription factors evolved to control the transcription of genes, in many cases, in response to external signals that are mediated by effectors [49]. Some transcription factors regulate the expression of a small number of genes, whilst others regulate hundreds [50]. Clearly, it is advantageous for bacteria to adapt to their surroundings, and this is especially true for the expression of virulence determinants that facilitate infection of mammalian hosts. Hence, in many bacterial pathogens, the expression of virulence factors, the proteins that are needed to establish and maintain successful infection, is regulated by one or more “dedicated” transcription factors [1,2]. These are often encoded by genes that are associated with plasmids or “pathogenicity islands” of genes encoding the virulence factors [1,2]. However, it is apparent that the expression of some genes that are essential for infection are regulated by “regular” transcription factors, whose activity is not restricted to infection situations [18,19,51]. This appears to be the case for expression of the *pic* mucinase gene in both EAEC 042 and UPEC CFT073.

The Pic mucinase is distributed widely in enteric bacteria [25,47,52]. The EAEC 042 *pic* allele and corresponding promoter region is found in *S. flexneri* and many EAEC strains, including the Shiga-toxin-producing EAEC O104:H4 strain C227–11, which carries two chromosomal copies of this variant (Figure S1) [25,53]. The, UPEC *picU* gene and promoter is found in many UPEC strains (*e.g., E. coli* strain ABU83972 and *E. coli* clone D i2) but also faecal isolates, including the well-known probiotic *E. coli* strain Nissle 1917 (Figure S1) [54–56]. As faecal *E. coli* are considered to be the major source of bacteria causing urinary tract infections, this is perhaps not surprising. However, it is of note that Nissle 1917 has many virulence determinants that are similar to UPEC isolates (Table S3). Many studies that have examined the distribution of Pic have not distinguished between the different Pic variants carried by bacteria (*i.e*., *pic* verses *picU*) and so it is unclear if *picU* is more likely to be carried by UPEC isolates [25,47,52].

Previously, Behrens *et al*. [57] investigated the EAEC 042 *pic* promoter and concluded that a single strong promoter was responsible for the majority of *pic* expression. Here we have reported the location of this promoter and the transcription factors involved in Pic regulation. The EAEC and UPEC *pic* promoters are unusual as they have evolved two precisely placed 10 hexamer elements, and, in the UPEC *pic*p073 fragment, both appear to be functional. Thus, the UPEC strain CFT073 *picU* promoter is the first example of what we dub as an ambiguous Class I/II CRP-dependent promoter. The advantage of this is still unclear, but data in Figure 4 show that *pic* gene translation levels differ according to which 5` transcript end is selected. Similarly, the factors that bias CRP-dependent activation in the *pic*p042 fragment towards the Class I option are unclear, but this study underscores that bacterial promoters can be active, whilst not conforming to simple textbook models. Interestingly, *Citrobacter rodentium* also carries the PicC mucinase, which has 79.15% and 78.57% identity to those from EAEC and UPEC, respectively (Figure S2) [58]. Comparison of the *picC* promoter with that from EAEC and UPEC suggests that it is likely CRP-dependent but a hybrid version of these two *E. coli* promoters, carrying the upstream of the UPEC *picU* promoter and the downstream region of the EAEC version (Figure S1). We surmise that the *C. rodentium pic* promoter functions by a Class II mechanism only, as the downstream −10 element is poor. Note that CRP in *C. rodentium*, and the *Enterobacteriaceae* in general, is highly conserved (Figure S3). Thus, it seems that evolution has produced three flavours of the *pic* promoter, CRP-dependent Class I only (EAEC), CRP-dependent Class II only (*C. rodentium picC*) and CRP-dependent Class I/II ambiguous (UPEC *picU*).

*E.coli* genomes encode over 300 transcription factors that support a complex regulatory network and, amongst the factors that make the most connections, are CRP and Fis [50] . CRP activity increases in response to nutrient stress, notably absence of glucose, whilst Fis levels rise during rapid growth [20,21,59,60]. Hence, genes whose products assure catabolic functions often carry promoters that are activated by CRP, whilst the promoters of genes whose products are not required during rapid growth are often repressed by Fis. In this light, it is easy to understand why the expression of bacterial mucinases should be regulated by both CRP and Fis, especially if ancestral mucinase genes encoded secreted proteinases with relaxed specificity for proteins other than mucin. Note that, in addition to mucin, Pic can degrade diverse substrates, including gelatine, complement proteins and human leukocyte surface glycoproteins [25,26,31,32] and that expression of both *pic* promoters is maximal in minimal medium (Figure S4). Presumably, this is part of a larger more general bacterial foraging response to cope with starvation and slow growth, and the ancestral gene was exapted in pathogens, such as EAEC and UPEC, specifically to facilitate mucin digestion, without its transcriptional regulation being altered. It is of note that the DNA surrounding the *pic* genes from EAEC 042, *S. flexneri* and UPEC CFT073 contains many genes from insertion sequences (Figure S5), suggesting that these genes are readily mobile and can be swapped between bacteria. As both CRP and Fis are found in all *Enterobacteriaceae*, should *pic* gene exchange occur, regulation will be maintained, perhaps highlighting the choice of global regulators over “bespoke” virulence transcription factors.

In addition to Pic, many pathogenic *Enterobacteriaceae* carry additional virulence genes, which encode other SPATE proteases, such as the Pet, SigA and Sat cytotoxins from EAEC 042, *S. flexneri* and UPEC CFT073, respectively [61,62]. In each case, toxin expression is activated by CRP, rather than a dedicated virulence transcription factor, but in this case CRP activation requires Fis as a co-activator, rather than a repressor. It has been argued that this ensures that toxin is released in specific conditions where bacterial growth and nutritional status is not too good, but not too bad and is resemblant of the “Goldilocks effect” [18,19]. Thus, the interplay between CRP and Fis appears to be recurrent theme in SPATE expression and is likely to be applicable to similar virulence determinants from other *Enterobacteriaceae*.

## Supplementary Material

Supplemental Material

## Data Availability

All data relating to this article are present in the article and the accompanying Supplementary Material.
