# Supplementary material for: Novel organisation and regulation of the pic promoter from enteroaggregative and uropathogenic Escherichia coli
Source: Virulence. 2022 Aug 16;13(1):1393–406. doi: 10.1080/21505594.2022.2111754 (PMC9387333; doi:10.1080/21505594.2022.2111754)
Supplement: Supplemental Material [file KVIR_A_2111754_SM5769.docx]

***For Virulence* Edited by DFB on 27/07/22**

**Novel organisation and regulation of the *pic* promoter from Enteroaggregative and Uropathogenic *Escherichia coli*.**

**Supplementary Material**

Munirah M. Alhammadi ^1,2#^, Rita E. Godfrey ^1#^, Joseph O. Ingram ^1^, Gurdamanjit Singh ^1^, Camilla L. Bathurst ^1^, Stephen J. W. Busby ^1^* and Douglas F. Browning ^1,3^*

^1^ Institute of Microbiology and Infection, School of Biosciences, University of Birmingham, Birmingham, B15 2TT, UK.

^2^ Biology Department, Princess Nourah bint Abdulrahman University, Riyadh 11671, Saudi Arabia.

^3^ College of Health & Life Sciences, Aston University, Aston Triangle, Birmingham, B4 7ET, UK.

^#^These authors contributed equally to ths work.

*To whom correspondence should be addressed:

Email: [s.j.w.busby@bham.ac.uk](mailto:s.j.w.busby@bham.ac.uk) Tel: +44 (0)121 414 5439

Email: [d.browning@aston.ac.uk](mailto:d.browning@aston.ac.uk)

**Table S1.** Strains, plasmids and promoter fragments used in this work.

**Bacterial strains. Relevant genotype or description. Reference or source.**

M182 *E. coli* Δ*lac* K-12 strain [[1](#_ENREF_1)]

M182 Δ*crp* Δ*crp* derivative of M182 [[1](#_ENREF_1)]

BW25113 *E. coli* K-12 *lacI*^q^, *rrnBT14,* *∆lacZ*_WJ16_ [[2](#_ENREF_2)]

*hsdR514,∆araBAD*_AH33,_ *∆rhaBAD*_LD78_.

BW25113 *crp::aph crp* replaced by a kanamycin resistance cassette. [[2](#_ENREF_2)]

BW25113 *fis::aph fis* replaced by a kanamycin resistance cassette. [[2](#_ENREF_2)]

EAEC 042 Wild type EAEC strain. [[3](#_ENREF_3)]

UPEC CFT073 Wild type UPEC strain. [[4](#_ENREF_4)]

**Bacterial plasmids.**

pSR pBR322 derivative containing a λ *oop* transcription [[5](#_ENREF_5)]

terminator and carries an ampicillin resistance cassette.

pRW224 A broad-host-range *lacZ* transcription fusion plasmid, [[6](#_ENREF_6)]

which allows cloning of promoter fragments as

transcriptional fusions to *lacZ* and carries a tetracycline

resistance cassette.

pRW225 A derivative of pRW224, which allows cloning of [[6](#_ENREF_6)]

promoter fragments as translational fusions to *lacZ*.

pJET1.2 A high copy number cloning vector carrying a ColE1 Thermo Fisher.

origin of replication and an ampicillin resistance cassette.

pD A pBR322 plasmid derivative carrying an ampicillin [[7](#_ENREF_7)]

resistance cassette.

pDCRP A pD derivative carrying the *crp* gene. [[7](#_ENREF_7)]

pDCRP AR1^-^ A pDCRP derivative carrying a substitution in CRP [[7](#_ENREF_7)]

activating region AR1 (HL159).

pDCRP AR2^-^  A pDCRP derivative carrying a substitution in CRP [[7](#_ENREF_7)]

activating region AR2 (KE101).

pDCRP AR1^-^ & 2^-^ A pDCRP derivative carrying substitutions in CRP [[7](#_ENREF_7)]

activating regions AR1 (HL159) and AR2 (KE101).

pPic pACYC184 derivative containing an EcoRI-ScaI [[8](#_ENREF_8)]

genomic fragment containing *pic* cloned from EAEC

strain 042, carries a tetracycline resistance cassette.

**Promoter fragments. All fragments are flanked by EcoRI and HindIII sites.**

*pic*p042 A 179 bp DNA fragment carrying the *pic* promoter This study

region from EAEC strain 042.

*pic*p042 p58A A derivative of *pic*p042 carrying a T to A substitution This study.

at position -58 upstream of the *pic* transcription start.

*pic*p042 p56C A derivative of *pic*p042 carrying an A to C substitution This study.

at position -56 upstream of the *pic* transcription start.

*pic*p042 p55T A derivative of *pic*p042 carrying a C to T substitution This study.

at position -55 upstream of the *pic* transcription start.

*pic*p042 p52G A derivative of *pic*p042 carrying an A to G substitution This study.

at position -52 upstream of the *pic* transcription start.

*pic*p042 p49+A A derivative of *pic*p042 carrying an A insertion at This study.

position -49 upstream of the *pic* transcription start.

*pic*p042 p31C A derivative of *pic*p042 carrying an A to C substitution This study.

at position -31 upstream of the *pic* transcription start.

*pic*p042 p14Cp11C A derivative of *pic*p042 carrying a G to C substitution This study.

at position -14 and A to C substitution at -11 upstream

of the *pic* transcription start.

*pic*p042 A derivative of *pic*p042 carrying substitutions at This study.

p31Cp14Cp11C positions -31, -14 and -11 upstream of the *pic* transcription

start.

*pic*p073 A 119 bp DNA fragment carrying the *pic* promoter This study

region from UPEC strain CFT073.

*pic*p073 p37A A derivative of *pic*p073 carrying a T to A substitution This study.

at position -37 upstream of the *pic* transcription start.

*pic*p073 p34T A derivative of *pic*p073 carrying a C to T substitution This study.

at position -34 upstream of the *pic* transcription start.

*pic*p073 p10C A derivative of *pic*p073 carrying an A to C substitution This study.

at position -10 upstream of the *pic* transcription start.

*pic*p073 p+8Cp+11C A derivative of *pic*p073 carrying a G to C substitution This study.

at position +8 and an A to C substitution at position

+11 downstream of the *pic* transcription start.

*pic*p073 A derivative of *pic*p073 carrying substitutions at This study.

p10Cp+8Cp+11C positions -10, +8 and +11.

**Table S2.** DNA Primers used in this work. Primers (all are shown 5' to 3').

**Primer name. Sequence ^a^.**

D10520 CCCTGCGGTGCCCCTCAAG

D10527 GCAGGTCGTTGAACTGAGCCTGAAATTCAG

SP1 CTGGCGAAAGGGGGATGTGCTGCAA

lacZRev GGCGATTAAGTTGGGTAACGCCAGGG

dT-anchor TTTTTTTTTTTTTTTTTV

pic042 EcoRI GGGGGAATTCGATCTGGCAGCCTGAGTTCACAG

pic042 HindIII GGGGGGAAGCTTATGATGTTTACAAGGGATATAAAAG

picCFT EcoRI GGGGGGAATTCGAAAAGTATTTCACTATGTAACAG

picCFT HindIII GGGGGAAGCTTATGATGTTTAAGTACTAATGATAACC

picCTF p37A GGGGGAATTCGAAAAGTATTTCACTATGTAACAGACAACACAAAAATAC

picCTF p34T GGGGGAATTCGAAAAGTATTTCACTATGTAACAGACATCATAAAAATAC

pic042-UKO CATTAATGCAGTAACTCTATTTTCC

pic073-UKO CGCCCGGGTCATCACGTCTGGTTATC

pic042-DKO CCTTTCTGGTCATCACGTCGGGTTATC

pic073-DKO CCGCCCGGGTCATCACGTCTGGTTATC

pic042-225 GGGGAAGCTTAACTTTATTCACTATGGATTCTCCATGATGTTTACAAGG

pic073-225 GGGGAAGCTTAA CTT TATTCACTGCGGACTCTCCATGATGTTTAAGTAC

^a^ Restriction sites are underlined.

**Table S3.** Genome analysis of *E. coli strains* CFT073, ABU83972, Nissle 1917, Clone D i2 and EAEC 042.

|  | **CFT073** | **ABU83972** | **Nissle 1917** | **Clone D i2** | **EAEC 042** |
| --- | --- | --- | --- | --- | --- |
| Phylotype | B2 | B2 | B2 | B2 | D |
| Sequence Type **^a^** | ST73 | ST73 | ST73 | ST73 | ST414 |
| Serotype **^b^** | O6:H1 | O25:H1 | O6:H1 | O6:H1 | O44:H18 |
| Virulence genes **^c^** | *cea, chuA, clbB, focCsfaE, focG, foci, fyuA, gad, iha, ireA, iroN, irp2, iss, iucC, iutA, kpsE, kpsMII, mchB, mchF, mcmA, ompT, papA_F7-1, papA_F7-2, papC,* ***picU,*** *sat, sitA, tcpC, terC, usp, vat, yfcV* | *cea, chuA, clbB, cnf1, focCsfaE, focG, foci, fyuA, gad, hra, iha, iroN, irp2, iss, iucC, iutA, kpsE, kpsMII_K5, mchB, mchC, mchF, mcmA, ompT, papA_F14, papA_F43, papC,* ***picU****, sat, sitA, terC, usp, vat, yfcV* | *cea, chuA, clbB, focCsfaE, focG, focI, fyuA, gad, iha, iroN, irp2, iss, iucC, iutA, kpsE, kpsMII_K5, mchB, mchC, mchF, mcmA, ompT, papA_F43,* ***picU****, sat, sitA, tcpC, terC, usp, vat, yfcV,* | *cea, chuA, clbB, focCsfaE, focG, focI, fyuA, gad, iha, iroN, irp2, iss, iucC, iutA, kpsE, kpsMII_K5, mchB, mchC, mchF, mcmA, ompT, papA_F43,* ***picU****, sat, sitA, tcpC, terC, usp, vat, yfcV* | ***orf3, orf4, aaiC, aap, aar, aatA, aafA, aafB, aafC, aafD, aggR, capU,*** *astA, air, eilA, gadA, gadB, chuA, fyuA, irp2, lpfA, mchB, mchC, mchF, mcmA, pet,* ***pic*** |

Software at the Center for Genomic Epidemiology (<http://www.genomicepidemiology.org/>) was used to identify: **^a^** the sequence type [[9](#_ENREF_9)], ^b^ the serotype [[10](#_ENREF_10)] and ^c^ the potential virulence genes [[11](#_ENREF_11)] of each strain. The genes in EAEC 042 that are activated by AggR are in bold [[12](#_ENREF_12), [13](#_ENREF_13)]. The *pic* and *picU* genes in all strains are highlighted in red.

**Supplementary Figure legends**

**Figure S1.** Alignment of *pic* promoters from various *E. coli* and *Shigella flexneri* strains. A) The panel shows a Clustal W alignment of the DNA sequences of the *pic* promoter from EAEC 042 (FN5554766.1), *S. flexneri* 2a 301 (NC_004337.2) and EAEC C227-11 (CP011331.1). B) The panel shows a Clustal W alignment of the DNA sequences of the *pic* promoter from UPEC CFT073 (NC_004431.1) with *E. coli* Nissle 1917 (NZ_CP022686.1), ABU83972 (NC_017631.1) and clone D i2 (NC_017651.1). C) The panel shows a Clustal W alignment of the DNA sequences of the *pic* promoter from EAEC 042 (FN5554766.1), UPEC CFT073 (NC_004431.1) and Citrobacter *rodentium* ICC168 plasmid pCROD1 (picC_Cr) (NC_013717.1). The CRP-binding site and -10 promoter elements are underlined, with matches to their respective consensus sequences in bold [[14](#_ENREF_14), [15](#_ENREF_15)]. Transcription start sites (+1) are lower case bold and translation initiation sites (GTG) are bold and underlined.

**Figure S2.** Alignment of the Pic proteins from EAEC 042, UPEC CFT073 and Citrobacter *rodentium* ICC168. The figure shows a Clustal W alignment of the amino acid sequences of the Pic mucinase from EAEC 042 (FN5554766.1), PicU from UPEC CFT073 (NC_004431.1) and PicC from Citrobacter *rodentium* ICC168 plasmid pCROD1 (NC_013717.1). The EAEC and UPEC Pic proteins have 96.5% identity. PicC has 79.15% and 78.57% identity with EAEC 042 Pic and UPEC CFT073 PicU, respectively.

**Figure S3.** Alignment of the CRP proteins from various Gram-negative bacteria. The figure shows a Clustal W alignment of the amino acid sequences of CRP from *E. coli* K-12 MG1655, EAEC 042, UPEC CFT073, S. flexneri 2a 301, enterohemorrhagic *E. coli* strain Sakai, Shigella boydii Sb227, *Salmonella Typhimurium* LT2, *Salmonella Typhi* CT8, *Salmonella Paratyphi* ATCC 9150, Escherichia fergusonii ATCC35469, Klebsiella pneumoniae 342, Proteus mirabilis HI4320, Yersinia pestis strain Angola, Yersinia pseudotuberculosis strain IP 31758 and Citrobacter rodentium ICC168. The DNA recognition helix of CRP and the location of AR1 (H159) and AR2 (K101) in the CRP protein from E. coli K-12 MG1655 is highlighted in yellow. Differences to the E. coli K-12 MG1655 CRP sequence are in red.

**Figure S4.** The EAEC and UPEC *pic* promoters are regulated in response to nutrient level. The figure displays β-galactosidase activities determined in *E. coli* strain BW25113, carrying the *lac* expression vector pRW224 into which the EAEC *pic*p042 and UPEC *pic*p073 promoter fragments were cloned. Cells were cultured in either minimal medium, LB medium or LB supplemented with 0.4 % glucose. β-galactosidase activities are stated as nmol of ONPG hydrolysed min^-1^ mg^‑1^ dry cell mass and are the average of at least three independent values. Standard deviations are indicated and * indicates P < 0.01 using a Student’s *t*-test.

**Figure S5**. Organisation of the chromosomal *pic* locus in EAEC 042, *S. flexneri* 2a 301 and UPEC CFT073. The figure shows the chromosomal *pic* locus and surrounding DNA in A) EAEC 042, B) *S. flexneri* 2a 301 and C) UPEC CFT073. Genomes were annotated and drawn using the software at the Proksee website (https://proksee.ca/projects/new) [[16](#_ENREF_16)]

**Supplementary Material References.**

[1] S. Busby, D. Kotlarz, H. Buc, Deletion mutagenesis of the Escherichia coli galactose operon promoter region, J Mol Biol. 167 (1983) 259-74.

[2] T. Baba, T. Ara, M. Hasegawa, Y. Takai, Y. Okumura, M. Baba, et al., Construction of *Escherichia coli* K-12 in-frame, single-gene knockout mutants: the Keio collection, Mol Syst Biol. 2 (2006) 2006 0008. doi: msb4100050 [pii]

10.1038/msb4100050.

[3] R.R. Chaudhuri, M. Sebaihia, J.L. Hobman, M.A. Webber, D.L. Leyton, M.D. Goldberg, et al., Complete genome sequence and comparative metabolic profiling of the prototypical enteroaggregative *Escherichia coli* strain 042, PLoS One. 5 (2010) e8801. doi: 10.1371/journal.pone.0008801.

[4] R.A. Welch, V. Burland, G. Plunkett, 3rd, P. Redford, P. Roesch, D. Rasko, et al., Extensive mosaic structure revealed by the complete genome sequence of uropathogenic *Escherichia coli*, Proc Natl Acad Sci U S A. 99 (2002) 17020-4. doi: 10.1073/pnas.252529799.

[5] A. Kolb, D. Kotlarz, S. Kusano, A. Ishihama, Selectivity of the *Escherichia coli* RNA polymerase E sigma 38 for overlapping promoters and ability to support CRP activation, Nucleic Acids Res. 23 (1995) 819-26.

[6] M.S. Islam, L.E. Bingle, M.J. Pallen, S.J. Busby, Organization of the LEE1 operon regulatory region of enterohaemorrhagic *Escherichia coli* O157:H7 and activation by GrlA, Mol Microbiol. 79 (2011) 468-83. doi: 10.1111/j.1365-2958.2010.07460.x.

[7] D. West, R. Williams, V. Rhodius, A. Bell, N. Sharma, C. Zou, et al., Interactions between the Escherichia coli cyclic AMP receptor protein and RNA polymerase at class II promoters, Mol Microbiol. 10 (1993) 789-97.

[8] S.M. Harrington, J. Sheikh, I.R. Henderson, F. Ruiz-Perez, P.S. Cohen, J.P. Nataro, The Pic protease of enteroaggregative *Escherichia coli* promotes intestinal colonization and growth in the presence of mucin, Infect Immun. 77 (2009) 2465-73. doi: IAI.01494-08 [pii]

10.1128/IAI.01494-08.

[9] M.V. Larsen, S. Cosentino, S. Rasmussen, C. Friis, H. Hasman, R.L. Marvig, et al., Multilocus sequence typing of total-genome-sequenced bacteria, J Clin Microbiol. 50 (2012) 1355-61. doi: 10.1128/jcm.06094-11.

[10] K.G. Joensen, A.M. Tetzschner, A. Iguchi, F.M. Aarestrup, F. Scheutz, Rapid and Easy *In Silico* Serotyping of *Escherichia coli* Isolates by Use of Whole-Genome Sequencing Data, J Clin Microbiol. 53 (2015) 2410-26. doi: 10.1128/jcm.00008-15.

[11] K.G. Joensen, F. Scheutz, O. Lund, H. Hasman, R.S. Kaas, E.M. Nielsen, et al., Real-time whole-genome sequencing for routine typing, surveillance, and outbreak detection of verotoxigenic *Escherichia coli*, J Clin Microbiol. 52 (2014) 1501-10. doi: 10.1128/jcm.03617-13.

[12] N. Morin, C. Tirling, S.M. Ivison, A.P. Kaur, J.P. Nataro, T.S. Steiner, Autoactivation of the AggR regulator of enteroaggregative *Escherichia coli in vitro* and *in vivo*, FEMS Immunol Med Microbiol. 58 (2010) 344-55. doi: 10.1111/j.1574-695X.2010.00645.x.

[13] M. Yasir, C. Icke, R. Abdelwahab, J.R. Haycocks, R.E. Godfrey, P. Sazinas, et al., Organization and architecture of AggR-dependent promoters from enteroaggregative *Escherichia coli*, Mol Microbiol. 111 (2019) 534-51. doi: 10.1111/mmi.14172.

[14] D.F. Browning, S.J. Busby, Local and global regulation of transcription initiation in bacteria, Nat Rev Microbiol. 14 (2016) 638-50. doi: 10.1038/nrmicro.2016.103.

[15] S. Busby, R.H. Ebright, Transcription activation by catabolite activator protein (CAP), J Mol Biol. 293 (1999) 199-213. doi: 10.1006/jmbi.1999.3161.

[16] J.R. Grant, P. Stothard, The CGView Server: a comparative genomics tool for circular genomes, Nucleic Acids Res. 36 (2008) W181-4. doi: 10.1093/nar/gkn179.

**Figure S1.**

**a)**

pic1_C227-11 ACAGATAAAACACTCTCCAGGAAACCCGGG**G**C**G**GTTCAGT**TCACA**AAAACACATTAATGC

pic2_C227-11 ACAGATAAAACACTCTCCAGGAAACCCGGG**G**C**G**GTTCAGT**TCACA**AAAACACATTAATGC

pic_Sf2a-301 ACAGATAAAACACTCTCCAGGAAACCCGGG**G**C**G**GTTCAGT**TCACA**AAAACACATTAATGC

pic_042 ACAGATAAAACAATCTCCAGGAAACCCGGG**G**C**G**GTTCAGT**TCACA**AAAACACATTAATGC

************ ***********************************************

**TGTGA TCACA**

pic1_C227-11 AGTAACTATATTTTCCTTTCTGGTGA**TA**ACG**T**CGGGTT**a**TCATTAGCTTCTTCAGCTATT

pic2_C227-11 AGTAACTATATTTTCCTTTCTGGTGA**TA**ACG**T**CGGGTT**a**TCATTAGCTTCTTCAGCTATT

pic_Sf2a-301 AGTAACTATATTTTCCTTTCTGGTGA**TA**ACG**T**CGGGTT**a**TCATTAGCTTCTTCAGCTATT

pic_042 AGTAACTATATTTTCCTTTCTGGTGA**TA**ACG**T**CGGGTT**a**TCATTAGCTTCTTCAGCTATT

************************************************************

**TATAAT +1**

pic1_C227-11 TTACTTTTATATCCCTTGTAAACATCATGGAGAATCCATA**GTG**AATAAAGTTTATTCTCT

pic2_C227-11 TTACTTTTATATCCCTTGTAAACATCATGGAGAATCCATA**GTG**AATAAAGTTTATTCTCT

pic_Sf2a-301 TTACTTTTATATCCCTTGTAAACATCATGGAGAATCCATA**GTG**AATAAAGTTTATTCTCT

pic_042 TTACTTTTATATCCCTTGTAAACATCATGGAGAATCCATA**GTG**AATAAAGTTTATTCTCT

************************************************************

***pic* 🡪**

**b)**

picU_CFT073 CAGGAGCGGTAATTTTGTAAGCAAACGAAAAGTATTTCACTA**TGT**A**A**CAGACA**TCACA**AA

pic_ABU83972 CAGGAGCGGTAATTTTGTAAGCAAACGAAAAGTATTTCACTA**TGT**A**A**CAGACA**TCACA**AA

pic_Nissle CAGGAGCGGTAATTTTGTAAGCAAACGAAAAGTATTTCACTA**TGT**A**A**CAGACA**TCACA**AA

pic_D_i2 CAGGAGCGGTAATTTTGTAAGCAAACGAAAAGTATTTCACTA**TGT**A**A**CAGACA**TCACA**AA

************************************************************

**TGTGA TCACA**

picU_CFT073 AATACATTAATGCAGTCAC**TATA**T**T**TTCCG**c**CCGGGTGATAACGTCTGGTTATCATTAGT

pic_ABU83972 AATACATTAATGCAGTCAC**TATA**T**T**TTCCG**c**CCGGGTGATAACGTCTGGTTATCATTAGT

pic_Nissle AATACATTAATGCAGTCAC**TATA**T**T**TTCCG**c**CCGGGTGATAACGTCTGGTTATCATTAGT

pic_D_i2 AATACATTAATGCAGTCAC**TATA**T**T**TTCCG**c**CCGGGTGATAACGTCTGGTTATCATTAGT

************************************************************

**TATAAT +1**

picU_CFT073 ACTTAAACATCATGGAGAGTCCGCA**GTG**AATAAAGTTTATTCTCT

pic_ABU83972 ACTTAAACATCATGGAGAGTCCGCA**GTG**AATAAAGTTTATTCTCT

pic_Nissle ACTTAAACATCATGGAGAGTCCGCA**GTG**AATAAAGTTTATTCTCT

pic_D_i2 ACTTAAACATCATGGAGAGTCCGCA**GTG**AATAAAGTTTATTCTCT

*********************************************

***picU* 🡪**

**c)**

picU_CFT073 TAATTTTGTAAGCAAACGAAAAGTATTTCACTA**TGT**A**A**CAGACA**TCACA**AAAATACATTA

pic_042 --ACAGATAAAACAATCTCCAGGAA--ACCCGGG**G**C**G**GTTCAGT**TCACA**AAAACACATTA

picC_Cr TAAAATAAAAACCAAATGTAACGATTTACCTTA**TGT**T**A**ACTGAG**TCACA**AAAAAGCAGTA

* ** *** * * * * * ********* ** **

**TGTGA TCACA**

picU_CFT073 ATGCAGTCAC**TATA**T**T**TTCCG**c**CCGGGTGA**TA**ACG**T**CTGGTTATCATTAG----------

pic_042 ATGCAGTAAC**TATA**T**T**TTCCTTTCTGGTGA**TA**ACG**T**CGGGTT**a**TCATTAGCTTC------

picC_Cr CATCATTCAC**TATA**T**T**CCCC-TCCTTGTGA**TA**ACTGCGGGTTATGGTTATCACTCAAGCA

** * ******** ** * ******** * ****** ***

**TATAAT +1**  **TATAAT +1**

picU_CFT073 -----------TACTT---------------AAACATCATGGAGAGTCCGCA**GTG**AATAA

pic_042 TTCAGCTATTTTACTTTT--ATATCCCTTGTAAACATCATGGAGAATCCATA**GTG**AATAA

picC_Cr TTTAGCTGATTTATTTTTTCATATTATCTGTGATTGTCATGGAGGGTTTGCT**GTG**AATAA

** ** * ******** * ********

***pic* 🡪**

**Figure S2.**

PicU_CFT073 MNKVYSLKYCPVTGGLIVVSELASRVIKKTCRRLTHILLAGIPAVYLYYPQISQAGIVRS

Pic_042 MNKVYSLKYCPVTGGLIAVSELARRVIKKTCRRLTHILLAGIPAICLCYSQISQAGIVRS

PicC_Cr MNKIYSLKYCPVTQGLIAVSELASRVIKKISRKLKSITLITFSVASLAYPAISQAGIVRA

***:********* ***.***** ***** .*:*. * * :.. * *. ********:

PicU_CFT073 DIAYQIYRDFAENKGLFVPGATDIPVYDKDGKLVGRLDKAPMADFSSVSSNGVATLVSPQ

Pic_042 DIAYQIYRDFAENKGLFVPGANDIPVYDKDGKLVGRLGKAPMADFSSVSSNGVATLVSPQ

PicC_Cr DIPYQLFRDFAENKGIFVPGAADIPVYDKDGKLVGHLDKAPMADFSSVTTNAVATLVSPQ

**.**::********:***** *************:*.**********::*.********

PicU_CFT073 YIVSVKHNGGYQSVSFGNGKNTYSLVDRNNHSSVDFHAPRLNKLVTEVIPSAITSEGTKA

Pic_042 YIVSVKHNGGYRSVSFGNGKNTYSLVDRNNHPSIDFHAPRLNKLVTEVIPSAVTSEGTKA

PicC_Cr YLVSVKHNGGYQTVSFGDGKNSYSLVDRNNHPSTDFHAPRLSKLVTEVIPASVTAEGTKR

*:*********::****:***:*********.* *******.********:::*:****

PicU_CFT073 NAYKDTERYTAFYRVGSGTQYTKDKDGNLVKVAGGYAFKTGGTTGVPLISDATIVSNPGQ

Pic_042 NAYKYTERYTAFYRVGSGTQYTKDKDGNLVKVAGGYAFKTGGTTGVPLISDATIVSNPGQ

PicC_Cr NAYKNTERYTEFYRVGSGTQYTKDRDGKLTKIAGGYAFKTGGTIGTPLISNGTIVTNPGQ

**** ***** *************:**:*.*:*********** *.****:.***:****

PicU_CFT073 TYNPVNGPLPDYGAPGDSGSPLFAYDEQQKKWVIVAVLRAYAGINGATNWWNVIPTDYLN

Pic_042 TYNPVNGPLPDYGAPGDSGSPLFAYDKQQKKWVIVAVLRAYAGINGATNWWNVIPTDYLN

PicC_Cr TFNPVNGLLPSYGVPGDSGSPLFAYDSLQKKWVIVGVLKSYEGLRGATSWWNVIPTDYLN

*:***** **.**.************. *******.**::* *:.***.***********

PicU_CFT073 QVMQDDFDAPVDFVSGLPPLNWTYDKTSGTGTLSQGSKNWTMHGQKDNDLNAGKNLVFSG

Pic_042 QVMQDDFDAPVDFVSGLGPLNWTYDKTSGTGTLSQGSKNWTMHGQKDNDLNAGKNLVFSG

PicC_Cr KVMEEDFDAPVAAVSGKEPLAWNFDKNTGTGTLSQGTTTWDMHGQKGKDLNAGKNLVFSG

:**::****** *** ** *.:**.:********:..* *****.:************

PicU_CFT073 QNGAIVLKDSVTQGAGYLEFKDSYTVSAESGKTWTGAGIITDKGTNVTWKVNGVAGDNLH

Pic_042 QNGAIILKDSVTQGAGYLEFKDSYTVSAESGKTWTGAGIITDKGTNVTWKVNGVAGDNLH

PicC_Cr QNGAILLKDSVTQGAGYLEFKDSYTVSADSGKTWTGAGIITDKGTDVTWKVNGVAGDNLH

*****:**********************:****************:**************

PicU_CFT073 KLGEGTLTINGTGVNPGGLKTGDGTVVLNQQADTAGNVQAFSSVNLASGRPTVVLGDARQ

Pic_042 KLGEGTLTINGTGVNPGGLKTGDGIVVLNQQADTAGNIQAFSSVNLASGRPTVVLGDARQ

PicC_Cr KLGEGTLTVNGSGVNPGGLKTGDGTVILAQKPDAAGNVQAFSSVNLASGRPTVVLTDSKQ

********:**:************ *:* *:.*:***:***************** *::*

PicU_CFT073 VNPDNISWGYRGGKLDLNGNAVTFTRLQAADYGAVITNNAQQKSRLLLDLKAQDTNVSVP

Pic_042 VNPDNISWGYRGGKLDLNGNAVTFTRLQAADYGAVITNNAQQKSQLLLDLKAQDTNVSEP

PicC_Cr VNPDNISWGYRGGVLDLNGNDITFTRLRVSDYGAVIANKAANKSHLSLNLSAANNEDVSV

************* ****** :*****:.:******:*:* :**:* *:*.* :.:

PicU_CFT073 IG-SISPFGGTGTPGNLYSMILNGQTRFYILKSASYGNTLWGNSLNDPAQWEFVGTDKNK

Pic_042 TIGNISPFGGTGTPGNLYSMILNSQTRFYILKSASYGNTLWGNSLNDPAQWEFVGMDKNK

PicC_Cr PIGTVNPFGGKGTPGSLYSRNLNGQTSYYILKSASYGNTLWGNSLNNPAEWEFVGHDKNK

.:.****.****.*** **.** :******************:**:***** ****

PicU_CFT073 AVQTVKDRILAGRAKQPVIFHGQLTGNMDVTIPQLPGGRKVILDGSVNLPEGTLSEDSGT

Pic_042 AVQTVKDRILAGRAKQPVIFHGQLTGNMDVAIPQVPGGRKVIFDGSVNLPEGTLSQDSGT

PicC_Cr AVQTVKDRVLAERAKQPVIYHGQLNGNMDVTIPQLPGERKVVMDGSVNLPGGNLSKEGGS

********:** *******:****.*****:***:** ***::******* *.**::.*:

PicU_CFT073 LIFQGHPVIHASVSGSAPVSLNQKDWENRQFIMKTLSLKDADFHLSRNASLNSDIKSDNS

Pic_042 LIFQGHPVIHASISGSAPVSLNQKDWENRQFTMKTLSLKDADFHLSRNASLNSDIKSDNS

PicC_Cr LIFQGHPVIHASVNGSAPVSLTQKDWETRQFTLKTLSLKNADFHLSRNAVLNGDIQADNS

************:.*******.*****.*** :******:********* **.**::***

PicU_CFT073 HITLGSDRVFVDKNDGTGNYVILEEGTSVPDTVNDRSQYEGNITLDHNSTLDIGSRFTGG

Pic_042 HITLGSDRAFVDKNDGTGNYVIPEEGTSVPDTVNDRSQYEGNITLNHNSALDIGSRFTGG

PicC_Cr HIALGSGKVFVDKNDSTGNYVIPEEGSSSP----VESQYLGNVSLQKGSTLDINSRFTGG

**:***.:.******.****** ***:* * .*** **::*::.*:***.******

PicU_CFT073 IEAYDSAVSITSPDVLLTAPGAFAGSSLTVHDGGHLTALNGLFSDGHIQAGKNSKITLSG

Pic_042 IDAYDSAVSITSPDVLLTAPGAFAGSSLTVHDGGHLTALNGLFSDGHIQAGKNGKITLSG

PicC_Cr IEAHDSQVNVTSPDALLQNSGVFVNSSLSVRDGGHLTAQKGLYSDGRVQIGKNGTLSLSG

*:*:** *.:****.** .*.*..***:*:******* :**:***::* ***..::***

PicU_CFT073 TPVKDTAN-QYAPAVYLTDGYDLTGDNATLEITRGAHASGDIHASAASTVTIGSDTPAEL

Pic_042 TPVKDTAN-QYAPAVYLTDGYDLTGDNAALEITRGAHASGDIHASAASTVTIGSDTPAEL

PicC_Cr TPEAGADNTWMPVLTYMTEGYDLTGDNATLDISQQAHVSGDIHATSPSTIRIGSENPGSV

** .: * . .*:*:*********:*:*:: **.******::.**: ***:.*..:

PicU_CFT073 ASAETTASAFAGSLLEGYNAAFNGAITGGRADVSMHNALWTLGGDSAIHTLTVRNSRISS

Pic_042 ASAETAASAFAGSLLEGYNAAFNGAITGGRADVSMHNALWTLGGDSAIHSLTVRNSRISS

PicC_Cr ASSVTPVLAAG--LYSGYNAAYYGAITGGKGNVSMNNGLWQLTGDSGINNLTARNSRVQS

**: *.. * . * .*****: ******:.:***:*.** * ***.*:.**.****:.*

PicU_CFT073 EGDRTFRTLTVNKLDATGSDFVLRTDLKNADKINVTEKATGSDNSLNVSFMKDPAQGQSL

Pic_042 EGDRTFRTLTVNKLDATGSDFVLRTDLKNADKINVTEKATGSDNSLNVSFMNNPAQGQAL

PicC_Cr EEKGAFRTLTVNSLDATGSDFVLRTDLKNADKISVTGKASGSDNTLNVSFMKNPVPGQSL

* . :*******.********************.** **:****:******::*. **:*

PicU_CFT073 NIPLVTAPAGTSAEMFKAGTRMIGFSRVTPTLHVDTSGGNTKWILDGFKAEADKAAAAKA

Pic_042 NIPLVTAPAGTSAEMFKAGTRVTGFSRVTPTLHVDTSGGNTKWILDGFKAEADKAAAAKA

PicC_Cr NIPLVSAPAGTAENVFKAGTRVTGFSRVTPTLHVDTSGGSTKWILDGFRAEADKAAAAKA

*****:*****: ::******: ****************.********:***********

PicU_CFT073 DSFMNAGYKNFMTEVNNLNKRMGDLRDTNGDAGAWARIMSGAGSADGGYSDNYTHVQVGF

Pic_042 DSFMNAGYKNFMTEVNNLNKRMGDLRDTNGDAGAWARIMSGAGSADGGYSDNYTHVQVGF

PicC_Cr DSFMNAGYKNFMTEVNNLNKRMGELRDTKGDAGAWARIMNGAGSADGGYSDNYTHVQVGF

***********************:****:**********.********************

PicU_CFT073 DKKHELDGVDLFTGVTMTYTDSSADSHAFSGKTKSVGGGLYASALFESGAYIDLIGKYIH

Pic_042 DKKHELDGVDLFTGVTMTYTDSSADSHAFSGKTKSVGGGLYASALFESGAYIDLIGKYIH

PicC_Cr DKKHALDGVDLFTGVTMTYTDSSADSHAFSGKTKSVGGGLYASALFNSGAYIDLIGKYIH

**** *****************************************:*************

PicU_CFT073 HDNDYTGNFAGLGTKHYNTHSWYAGAETGYRYHLTEETFIEPQAELVYGAVSGKTFRWKD

Pic_042 HDNDYTGNFASLGTKHYNTHSWYAGAETGYRYHLTEDTFIEPQAELVYGAVSGKTFRWKD

PicC_Cr HDNDYTGNFAGLGTKHYGTHSWYAGAETGYRYHLTEDTFIEPQAELVYGAVSGKTFRWKD

**********.******.******************:***********************

PicU_CFT073 GDMDLSMKNRDFSPLIGRTGIELGKTFSGKDWSVTARAGTSWQFDLLNNGETVLRDASGE

Pic_042 GDMDLSMKNRDFSPLVGRTGVELGKTFSGKDWSVTARAGTSWQFDLLNNGETVLRDASGE

PicC_Cr GDMDLSMKNKDFSPLIGRTGVELGKTFSGKDWSVTARAGTSWQFDLLNNGETVLRDASGE

*********:*****:****:***************************************

PicU_CFT073 KRIKGEKDSRMLFNVGMNAQIKDNMRFGLEFEKSAFGKYNVDNAVNANFRYMF

Pic_042 KRIKGEKDSRMLFNVGMNAQIKDNMRFGLEFEKSAFGKYNVDNAVNANFRYMF

PicC_Cr KRIKGEKDSRMLFNVGMNAQIKDNIRVGLEFEKSAFGKYNVDNAINANFRYMF

************************:*.*****************:********

**Figure S3.**

CRP_S.Typhimurium MVLGKPQTDPTLEWFLSHCHIHKYPSKSTLIHQGEKAETLYYIVKGSVAV

CRP_K.pneumoniae MVLGKPQTDPTLEWFLSHCHIHKYPSKSTLIHQGEKAETLYYIVKGSVAV

CRP_C.rodentium MVLGKPQTDPTLEWFLSHCHIHKYPSKSTLIHQGEKAETLYYIVKGSVAV

CRP_S.Typhi _ MVLGKPQTDP**A**LEWFLSHCHIHKYPSKSTLIHQGEKAETLYYIVKGSVAV

CRP_S.Paratyphi MVLGKPQTDPTLEWFLSHCHIHKYPSKSTLIHQGEKAETLYYIVKGSVAV

CRP_S.boydii MVLGKPQTDPTLEWFLSHCHIHKYPSKSTLIHQGEKAETLYYIVKGSVAV

CRP_S.flexneri MVLGKPQTDPTLEWFLSHCHIHKYPSKSTLIHQGEKAETLYYIVKGSVAV

CRP_E.fergusonii MVLGKPQTDPTLEWFLSHCHIHKYPSKSTLIHQGEKAETLYYIVKGSVAV

CRP_EHEC MVLGKPQTDPTLEWFLSHCHIHKYPSKSTLIHQGEKAETLYYIVKGSVAV

CRP_EAEC MVLGKPQTDPTLEWFLSHCHIHKYPSKSTLIHQGEKAETLYYIVKGSVAV

CRP_UPEC MVLGKPQTDPTLEWFLSHCHIHKYPSKSTLIHQGEKAETLYYIVKGSVAV

CRP_K-12 MVLGKPQTDPTLEWFLSHCHIHKYPSKSTLIHQGEKAETLYYIVKGSVAV

CRP_Y.pestis MVLGKPQTDPTLEWFLSHCHIHKYPSKSTLIHQGEKAETLYYIVKGSVAV

CRP_Y.pseudotuberculosis MVLGKPQTDPTLEWFLSHCHIHKYPSKSTLIHQGEKAETLYYIVKGSVAV

CRP_P.mirabilis MVLGKPQTDPTLEWFLSHCHIHKYPSKSTLIHQGEKAETLYYIVKGSVAV

**********:***************************************

CRP_S.Typhimurium LIKDEEGKEMILSYLNQGDFIGELGLFEEGQERSAWVRAKTACEVAEISY

CRP_K.pneumoniae LIKDEEGKEMILSYLNQGDFIGELGLFEEGQERSAWVRAKTACEVAEISY

CRP_C.rodentium LIKDEEGKEMILSYLNQGDFIGELGLFEEGQERSAWVRAKTACEVAEISY

CRP_S.Typhi LIKDEEGKEMILSYLNQGDFIGELGLFEEGQERSAWVRAKTACEVAEISY

CRP_S.Paratyphi LIKDEEGKEMILSYLNQGDFIGELGLFEEGQERSAWVRAKTACEVAEISY

CRP_S.boydii LIKDEEGKEMILSYLNQGDFIGELGLFEEGQERSAWVRAKTACEVAEISY

CRP_S.flexneri LIKDEEGKEMILSYLNQGDFIGELGLFEEGQERSAWVRAKTACEVAEISY

CRP_E.fergusonii LIKDEEGKEMILSYLNQGDFIGELGLFEEGQERSAWVRAKTACEVAEISY

CRP_EHEC LIKDEEGKEMILSYLNQGDFIGELGLFEEGQERSAWVRAKTACEVAEISY

CRP_EAEC LIKDEEGKEMILSYLNQGDFIGELGLFEEGQERSAWVRAKTACEVAEISY

CRP_UPEC LIKDEEGKEMILSYLNQGDFIGELGLFEEGQERSAWVRAKTACEVAEISY

CRP_K-12 LIKDEEGKEMILSYLNQGDFIGELGLFEEGQERSAWVRAKTACEVAEISY

CRP_Y.pestis LIKDEEGKEMILSYLNQGDFIGELGLFEEGQERSAWVRAKTACEVAEISY

CRP_Y.pseudotuberculosis LIKDEEGKEMILSYLNQGDFIGELGLFEEGQERSAWVRAKTACEVAEISY

CRP_P.mirabilis LIKDEEGKEMILSYLNQGDFIGELGLFEE**D**QERSAWVRAKTACEVAEISY

*****************************.********************

**AR1 K101**

CRP_S.Typhimurium KKFRQLIQVNPDILMRLS**S**QMARRLQVTSEKVGNLAFLDVTGRIAQTLLN

CRP_K.pneumoniae KKFRQLIQVNPDILMRLS**S**QMARRLQVTSEKVGNLAFLDVTGRIAQTLLN

CRP_C.rodentium KKFRQLIQVNPDILMRLS**S**QMARRLQVTSEKVGNLAFLDVTGRIAQTLLN

CRP_S.Typhi _ KKFRQLIQVNPDILMRLS**S**QMARRLQVTSEKVGNLAFLDVTGRIAQTLLN

CRP_S.Paratyphi KKFRQLIQVNPDILMRLS**S**QMARRLQVTSEKVGNLAFLDVTGRIAQTLLN

CRP_S.boydii KKFRQLIQVNPDILMRLSAQMARRLQVTSEKVGNLAFLDVTGRIAQTLLN

CRP_S.flexneri KKFRQLIQVNPDILMRLSAQMARRLQVTSEKVGNLAFLDVTGRIAQTLLN

CRP_E.fergusonii KKFRQLIQVNPDILMRLSAQMARRLQVTSEKVGNLAFLDVTGRIAQTLLN

CRP_EHEC KKFRQLIQVNPDILMRLSAQMARRLQVTSEKVGNLAFLDVTGRIAQTLLN

CRP_EAEC KKFRQLIQVNPDILMRLSAQMARRLQVTSEKVGNLAFLDVTGRIAQTLLN

CRP_UPEC KKFRQLIQVNPDILMRLSAQMARRLQVTSEKVGNLAFLDVTGRIAQTLLN

CRP_K-12 KKFRQLIQVNPDILMRLSAQMARRLQVTSEKVGNLAFLDVTGRIAQTLLN

CRP_Y.pestis KKFRQLIQVNPDILMRLS**S**QMA**N**RLQ**I**TSEKVGNLAFLDVTGRIAQTLLN

CRP_Y.pseudotuberculosis KKFRQLIQVNPDILMRLS**S**QMA**N**RLQ**I**TSEKVGNLAFLDVTGRIAQTLLN

CRP_P.mirabilis KKFRQLIQVNPDILMRLSAQMA**N**RLQ**T**TSEKVGNLAFLDVTGRIAQTLLN

******************:***.*** ***********************

**AR1 H159 Recognition Helix**

CRP_S.Typhimurium LAKQPDAMTHPDGMQIKITRQEIGQIVGCSRETVGRILKMLEDQNLISAH

CRP_K.pneumoniae LAKQPDAMTHPDGMQIKITRQEIGQIVGCSRETVGRILKMLEDQNLISAH

CRP_C.rodentium LAKQPDAMTHPDGMQIKITRQEIGQIVGCSRETVGRILKMLEDQNLISAH

CRP_S.Typhi LAKQPDAMTHPDGMQIKITRQEIGQIVGCSRETVGRILKMLEDQNLISAH

CRP_S.Paratyphi LAKQPDAMTHPDGMQIKITRQEIGQI**A**GCSRETVGRILKMLEDQNLISAH

CRP_S.boydii LAKQPDAMTHPDGMQIKITRQEIGQIVGCSRETVGRILKMLEDQNLISAH

CRP_S.flexneri LAKQPDAMTHPDGMQIKITRQEIGQIVGCSRETVGRILKMLEDQNLISAH

CRP_E.fergusonii LAKQPDAMTHPDGMQIKITRQEIGQIVGCSRETVGRILKMLEDQNLISAH

CRP_EHEC LAKQPDAMTHPDGMQIKITRQEIGQIVGCSRETVGRILKMLEDQNLISAH

CRP_EAEC LAKQPDAMTHPDGMQIKITRQEIGQIVGCSRETVGRILKMLEDQNLISAH

CRP_UPEC LAKQPDAMTHPDGMQIKITRQEIGQIVGCSRETVGRILKMLEDQNLISAH

CRP_K-12 LAKQPDAMTHPDGMQIKITRQEIGQIVGCSRETVGRILKMLEDQNLISAH

CRP_Y.pestis LAKQPDAMTHPDGMQIKITRQEIGQIVGCSRETVGRILKMLEDQNLISAH

CRP_Y.pseudotuberculosis LAKQPDAMTHPDGMQIKITRQEIGQIVGCSRETVGRILKMLEDQNLISAH

CRP_P.mirabilis LAKQPDAMTHPDGMQIKITRQEIGQIVGCSRETVGRILKMLEDQNLISAH

**************************.***********************

CRP_S.Typhimurium GKTIVVYGTR

CRP_K.pneumoniae GKTIVVYGTR

CRP_C.rodentium GKTIVVYGTR

CRP_S.Typhi GKTIVVYGTR

CRP_S.Paratyphi GKTIVVYGTR

CRP_S.boydii GKTIVVYGTR

CRP_S.flexneri GKTIVVYGTR

CRP_E.fergusonii GKTIVVYGTR

CRP_EHEC GKTIVVYGTR

CRP_EAEC GKTIVVYGTR

CRP_UPEC GKTIVVYGTR

CRP_K-12 GKTIVVYGTR

CRP_Y.pestis GKTIVVYGTR

CRP_Y.pseudotuberculosis GKTIVVYGTR

CRP_P.mirabilis GKTIVVYGTR

**********

**Figure S4.**


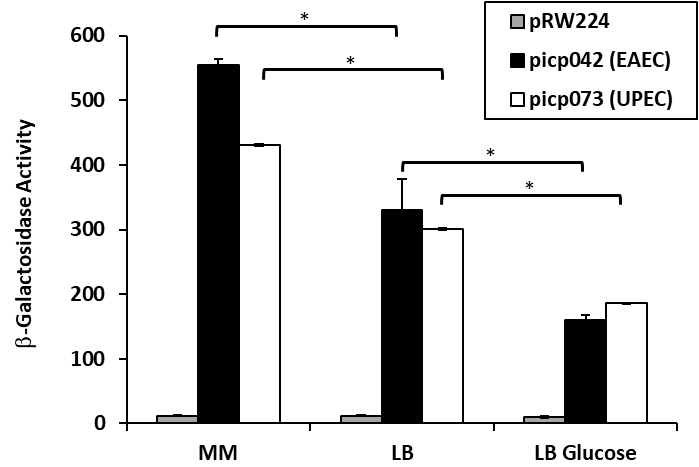


**
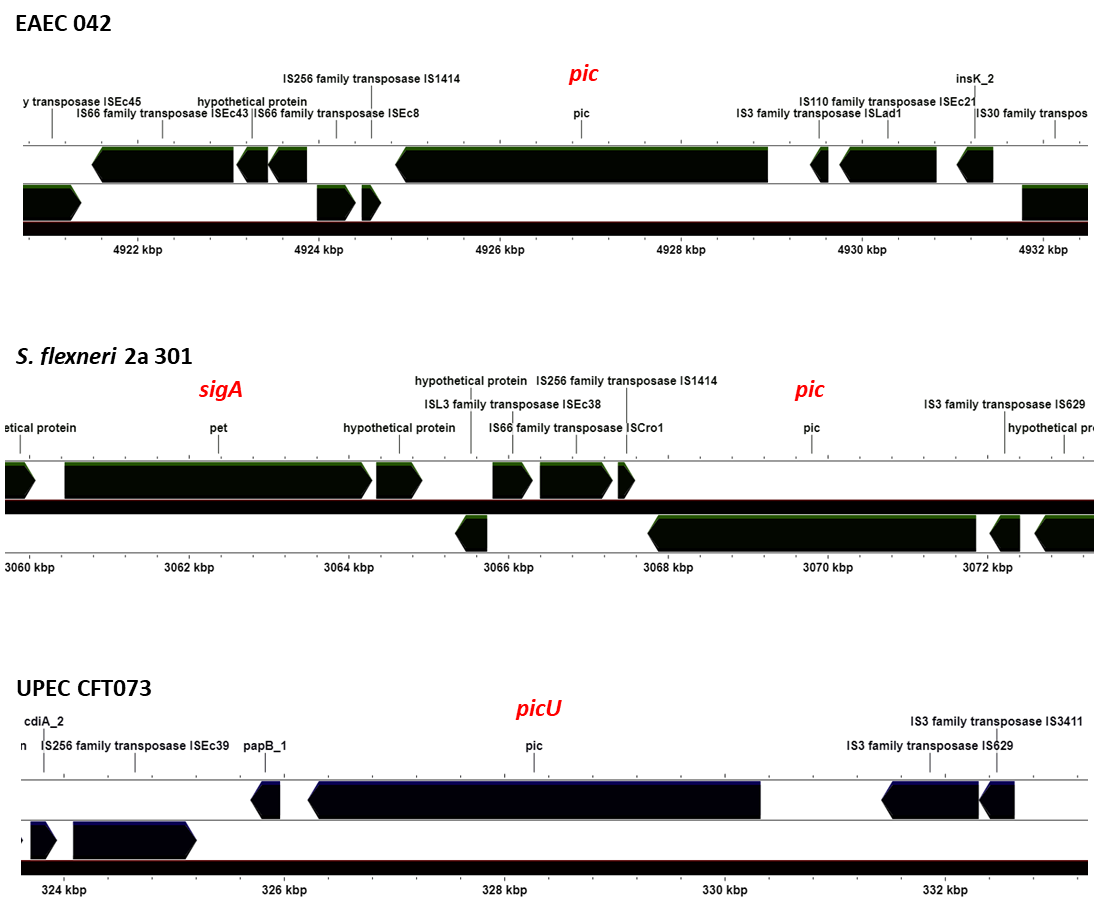
Figure S5.**
